# Global Scientific Trends in the Use of Mesenchymal Stem Cells for the Treatment of Diabetes Mellitus from 2000 to 2023: A Bibliometric Study

**DOI:** 10.2174/0118715303348724250310082224

**Published:** 2025-04-07

**Authors:** Zhenquan Sun, Yinghao Wang, Kexin Chen, Wen Zheng, Yang Wang, Mengxue Zhu, Min Yang, Xueyan Zhou

**Affiliations:** 1 Jiangsu Key Laboratory of New Drug Research and Clinical Pharmacy, Xuzhou Medical University, Xuzhou 221004, China;; 2 The First People’s Hospital of Changzhou, Changzhou 213003, China;; 3 NHC Key Laboratory of Nuclear Medicine, Jiangsu Key Laboratory of Molecular Nuclear Medicine, Jiangsu Institute of Nuclear Medicine, Wuxi 214063, China

**Keywords:** MSCs, diabetes, bibliometrics, citeSpace, vOSviewer, diabetic nephropathy

## Abstract

**Background:**

Diabetes mellitus is a chronic disease that seriously endangers human health, and mesenchymal stem cell (MSC) therapy is an emerging medical approach. Therefore, analyzing the effectiveness of MSCs in the literature is highly important for building relevant knowledge networks and promoting development in this field.

**Methods:**

The Web of Science database was selected as the source of literature, and all the literature on MSCs in diabetes from January 1, 2000, to October 18, 2023, was selected. After screening, CiteSpace and VOSviewer were imported into bibliometric software to construct the knowledge visualization map. Journal analysis, author analysis, country/region analysis, reference analysis, and keyword analysis were performed.

**Results:**

A total of 2912 articles and reviews were included. The number of articles on the use of MSCs for the treatment of diabetes is increasing annually. These publications originate mainly from 90 countries and 278 institutions, of which China and the United States were the top producers. We identified 15384 authors, with Liu Jiejie having the largest number of articles and Shapiro Amj being the most frequently co-cited. Stem Cell Research and Therapy was the most studied journal, and Diabetes was the most frequently cited journal. After analysis, the most common keywords were MSCs, diabetes mellitus, expression, transplantation, and differentiation.

**Conclusion:**

Research into MSC-based interventions for diabetes is booming. In the future, cooperation and connections between various countries and institutions should be strengthened. MSC-induced differentiation into insulin-producing cells, MSCs homing *in vivo*, the therapeutic effect of MSCs on diabetic nephropathy, and the therapeutic effect of exosomes secreted by MSCs constitute the current research hotspots and development trends for future research.

## 1 INTRODUCTION

Diabetes is a chronic metabolic disease characterized by generally elevated blood sugar levels and is often accompanied by various complications, including retinopathy, vascular disease, kidney disease, stroke, and foot ulcers, which can ultimately lead to multiple organ dysfunctions and failure. The causes of diabetes are very complex, including but not limited to environmental, lifestyle, and genetic factors. The majority (95%) of diabetes patients suffer from type 2 diabetes, and a small percentage have type 1 diabetes [1]. According to statistics, in the past 25 years, the incidence of diabetes in men has doubled, and in women, it has increased by 60% [2]. Noncommunicable diseases (NCDs) are responsible for 70% of global deaths, and the four leading NCDs are diabetes, cardiovascular disease, cancer, and respiratory diseases. Diabetes increases the risk of death in patients with the other three diseases by 30%-130% [3]. Therefore, diabetes has become a serious disease that critically affects the quality of life of people around the world.

Commonly used drugs for the treatment of diabetes include metformin, pioglitazone, exenatide, secretagogues, and insulin [4]. However, an increasing number of researchers have shown that drugs commonly used to treat diabetes can cause various serious side effects. For example, rosiglitazone can increase the risk of myocardial infarction in patients [5]. Pioglitazone has been found to have a protective effect on the cardiovascular system, but it may increase the risk of bladder cancer in men. As a result, pioglitazone is banned in France and Germany [6, 7]. The use of insulin glargine in research has been shown to increase the risk of cancer [8, 9]. Although these traditional small-molecule drugs can alleviate diabetes, they also have many accompanying side effects. Therefore, there is an urgent need for new drugs in the field of diabetes to improve diabetes treatment efficacy.

Mesenchymal Stem Cells (MSCs) are adult stem cells derived from the mesoderm that can differentiate into other tissues. They have a wide range of sources, including adipose tissue, bone marrow, and umbilical cord blood, among others [10]. With the development of regenerative medicine and cell therapy, MSCs have shown good therapeutic effects on diseases such as the treatment of ischemic cardiomyopathy by improving endothelial function [11], improvement of liver cirrhosis by secreting hepatocyte growth factor [12] and treatment of femoral head necrosis by promoting osteogenesis and angiogenesis, reducing adipogenesis [13]. The use of MSCs in the treatment of diabetes has been extensively studied by many researchers. In this study, literature analysis software was used to analyze the research on MSC intervention in diabetes in studies published in the 21^st^ century to determine the current research progress and possible future directions.

## 2 METHODS

### 2.1 Data Collection

**Fig. (1):**
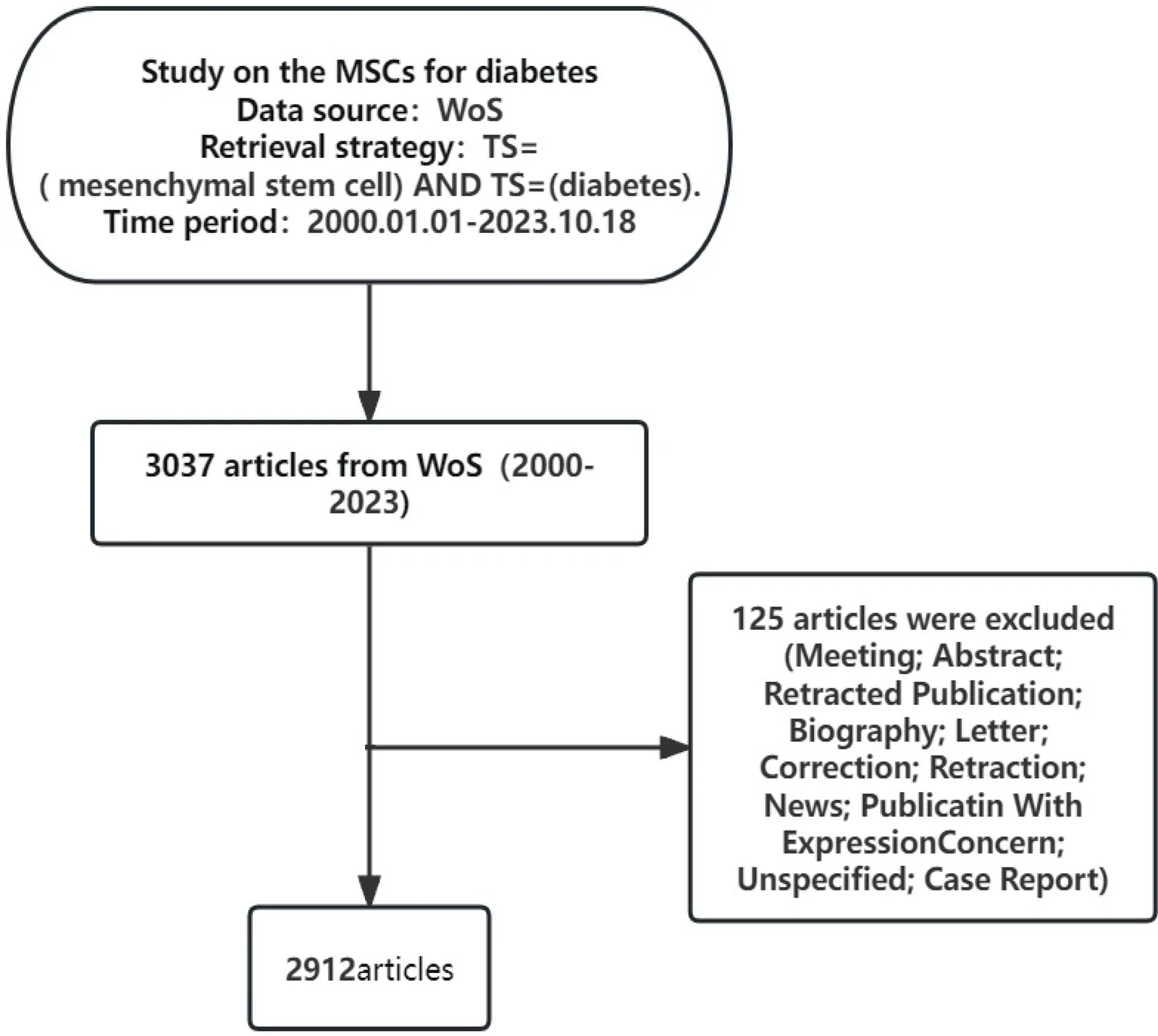
Flowchart of literature selection.

The data were retrieved and downloaded from the Web of Science (WoS) on October 26, 2023, using the following formula: TS=(mesenchymal stem cell) AND TS=(diabetes). We selected all research articles and reviews written in English from the beginning of the 21^st^ century to the retrieval date and then conducted screening (Fig. **1**). Finally, we obtained 2912 relevant studies. The literature was downloaded in full-record plain text format and named “download*.text” due to the use of the bibliometric software CiteSpace and VOSviewer.

### 2.2 Data Analysis

For data analysis and visualization, we selected CiteSpace and VOSviewer, two bibliometric software programs.

CiteSpace is a software tool that includes bibliometrics and visualization and is suitable for research collaborations between fields, connections between keywords, and potential development trends [14]. We used CiteSpace 6.2.R6 for studying the dual-map overlay of journals, visualizing countries/regions and institutions, visualizing keyword analysis, and analyzing co-cited references. The CiteSpace parameters were set as follows: period, 2000.01.01-2023.10.26; year per slice, 1; inclusive standards, top N = 50; and default values for other parameters.

VOSviewer has better performance in information-intensive visualization and image rendering [15]. We used VOSviewer for visualization analysis of journals and co-cited journals, authors, and co-cited authors.

### 2.3 Significance Statement

In this study, we used CiteSpace and VOSviewer software for the first time to construct a knowledge map of MSC intervention in diabetes, explore its research hotspots and development trends, and provide preliminary guidance for future researchers in this field.

## 3 RESULTS

### 3.1 The Trend of Publication Outputs

The number of published articles each year objectively reflects the development trend of research in this field. We collected a total of 2912 articles from 2000 to 2023, and the trend of the number of publications is shown in Fig. (**2**). Aside from the articles not yet published in 2023, the overall publication volume in this field shows a fluctuating upward trend every year. From 2000 to 2004, the article output was relatively low, indicating that many gaps in knowledge were waiting to be explored. From 2005 to the present, this number has experienced rapid growth, reaching its peak of 325 articles in 2021. This suggests that with the advancement of technology and theoretical innovations, interventions involving MSCs for diabetes treatment have attracted increasing amounts of attention.

### 3.2 Journals and Cocited Academic Journals

**Fig. (3):**
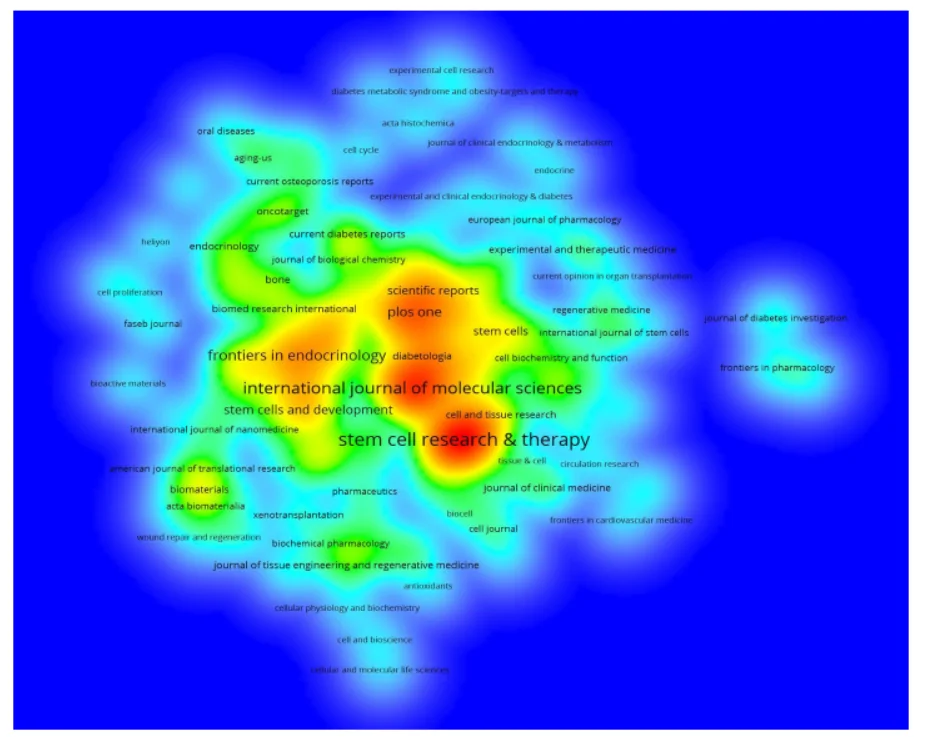
Density map of journals reporting on the use of MSCs for diabetes treatment (T≥5). **Note:** The word size, circle size, and opacity of red are associated with higher co-occurrence frequencies.

We used VOSviewer to analyze the publication journals and cocitation journals to explore the most representative and authoritative journals. A total of 2912 articles were retrieved from 841 different journals, and Stem Cell Research & Therapy (IF=7.5) was the most common (N=112, 3.84%), indicating that this journal has a significant impact on this field. The next highest number of articles were found in the International Journal of Molecular Sciences (N=85, IF=5.6) and Frontiers in Endocrinology (N=50, IF=5.2) (Table **1**, Fig. **3**). Among the top ten ranked journals, six were from the United States, and the rest were from Switzerland and the United Kingdom. The publications in the top ten journals account for 16% of the total publications, and half of the journals are in Q1 (JCR). Four journals had IFs greater than 5.

**Fig. (4):**
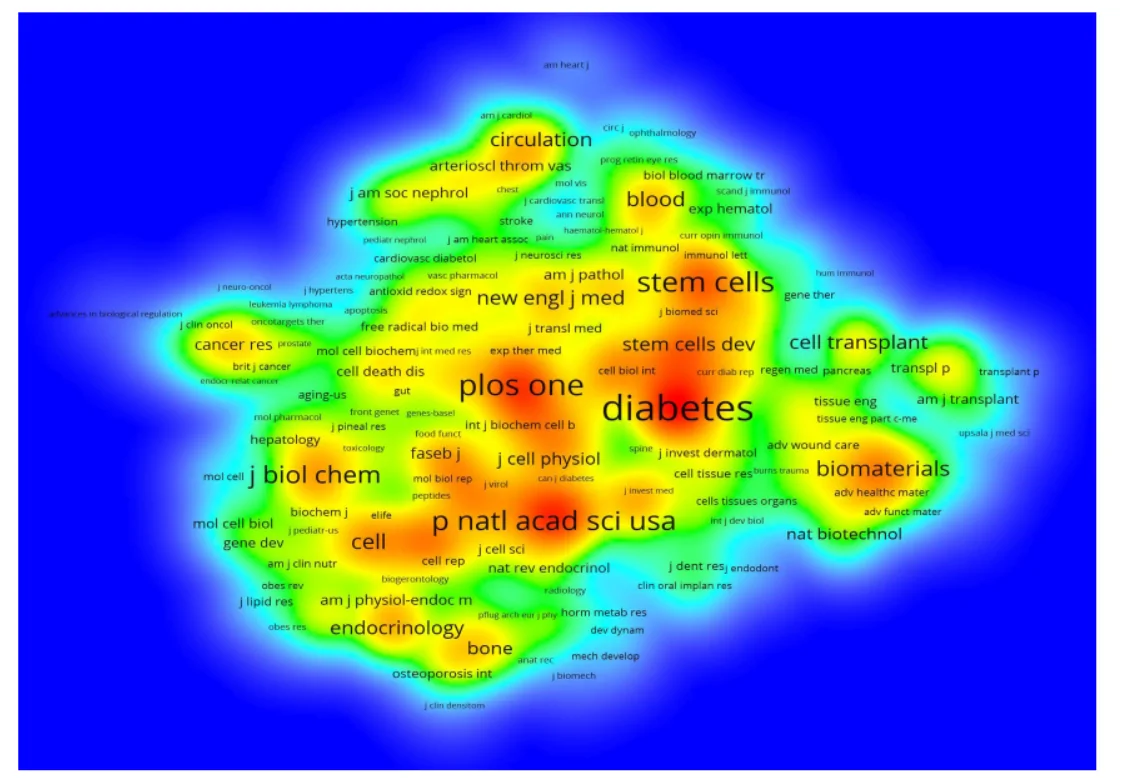
Density map of cocited journals reporting on the use of MSCs for diabetes treatment (T≥10). **Note:** The word size, circle size, and opacity of red are associated with higher co-occurrence frequencies.

The analysis of co-cited journals (Table **2**) revealed that the most commonly cited journal was Diabetes (N=6952, IF=7.7). Next are PLoS One (N=4457 IF=3.7), Stem Cells (N=4049 IF=5.2), Proceeding of the National Academy of Sciences of the United States of America (N=3713 IF=11.1), and Nature (N=3037 IF=64.8). Among the top ten cited journals, 5 journals were cited more than 3000 times, with Diabetes having a much higher citation rate than the other journals. A density map of co-cited journals (Fig. **4**) can more intuitively reflect the frequency of citation of the referenced journal. There were 3 journals with an IF greater than 10. The highest IF was found for Nature (IF=64.8), which was followed by Journal of Clinical Investigation (IF=15.9) and Proceeding of the National Academy of Science of the United States of America (IF=11.1).

**Fig. (5):**
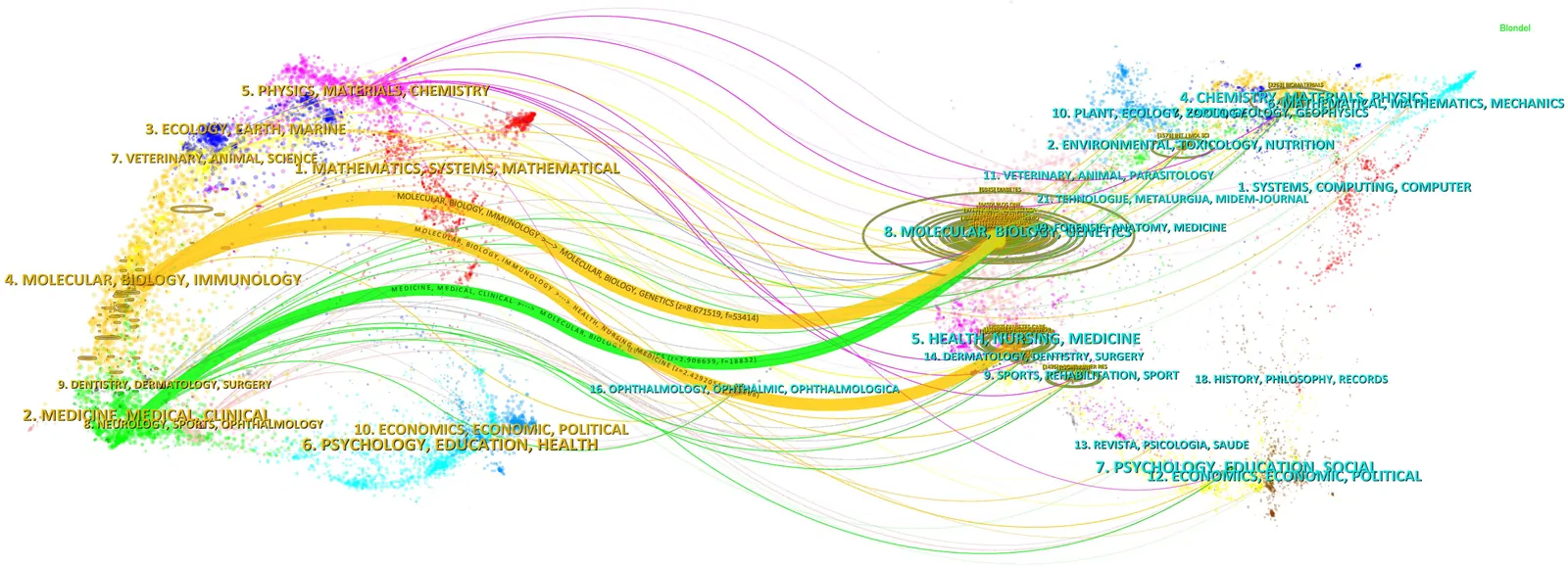
The dual-map overlay of journals reporting on the use of MSCs for diabetes treatment. **Note:** Citing journals are on the left, and cited journals are on the right. The colored paths between them suggest the cited relationships.

We used CiteSpace 6.2.R6 for a dual-map overlay of journals (Fig. **5**) to better reflect the distribution of journals and the relationships between journals. Three main reference paths were identified. Articles published in journals related to “Molecular biology and genetics” are frequently cited by articles in the field of “molecular biology, and immunology”. Articles published in journals related to “Health, nursing, and medicine” are often cited by articles in the field of “molecular biology, and immunology”. Articles published in journals related to “molecular, biology, and genetics” are often cited by articles in the field of “medicine, medicine, clinical.”.

### 3.3 Countries/Regions and Institutions

**Fig. (6):**
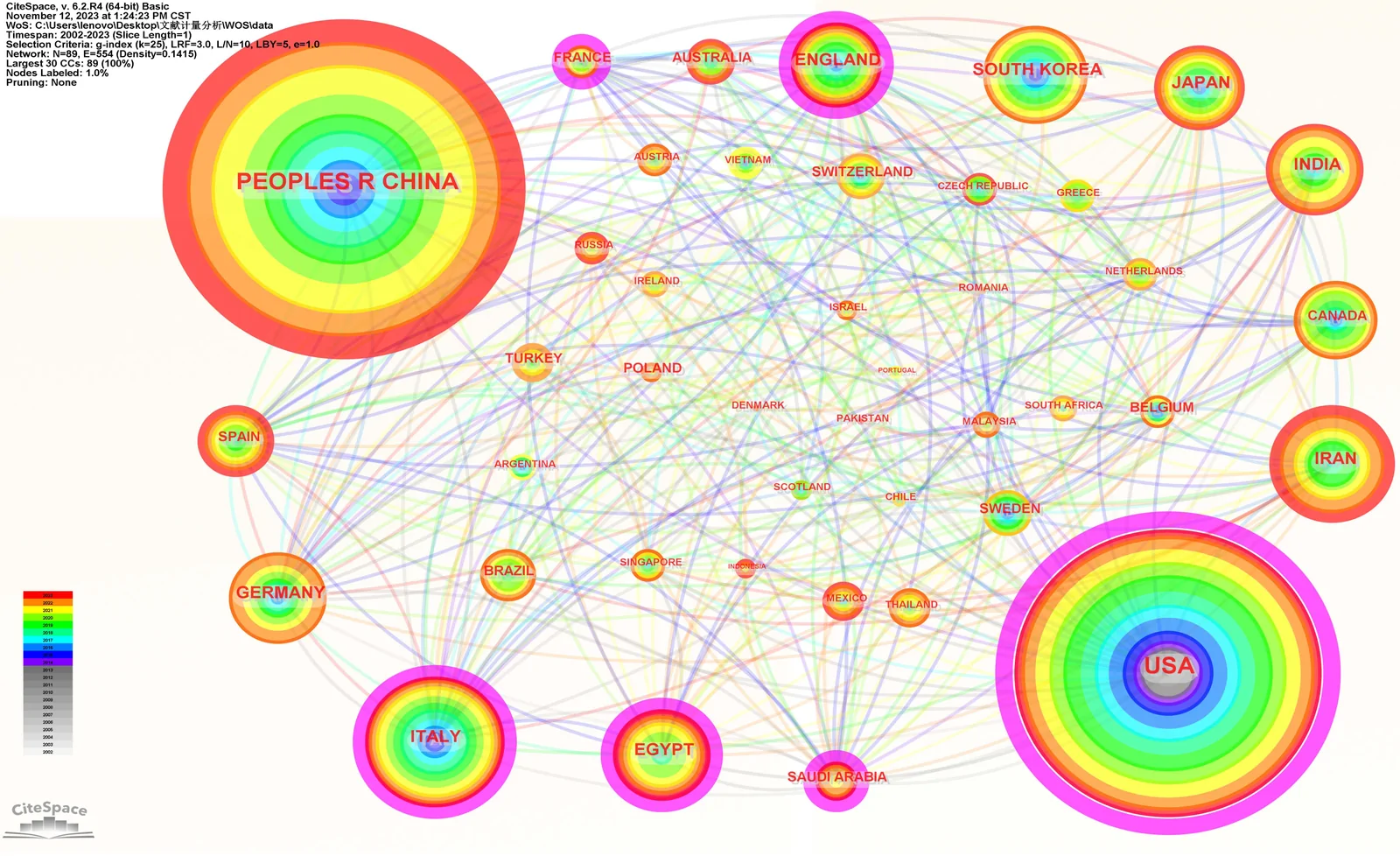
The co-occurrence map of countries/regions in which MSCs are used to treat diabetes.

**Fig. (7):**
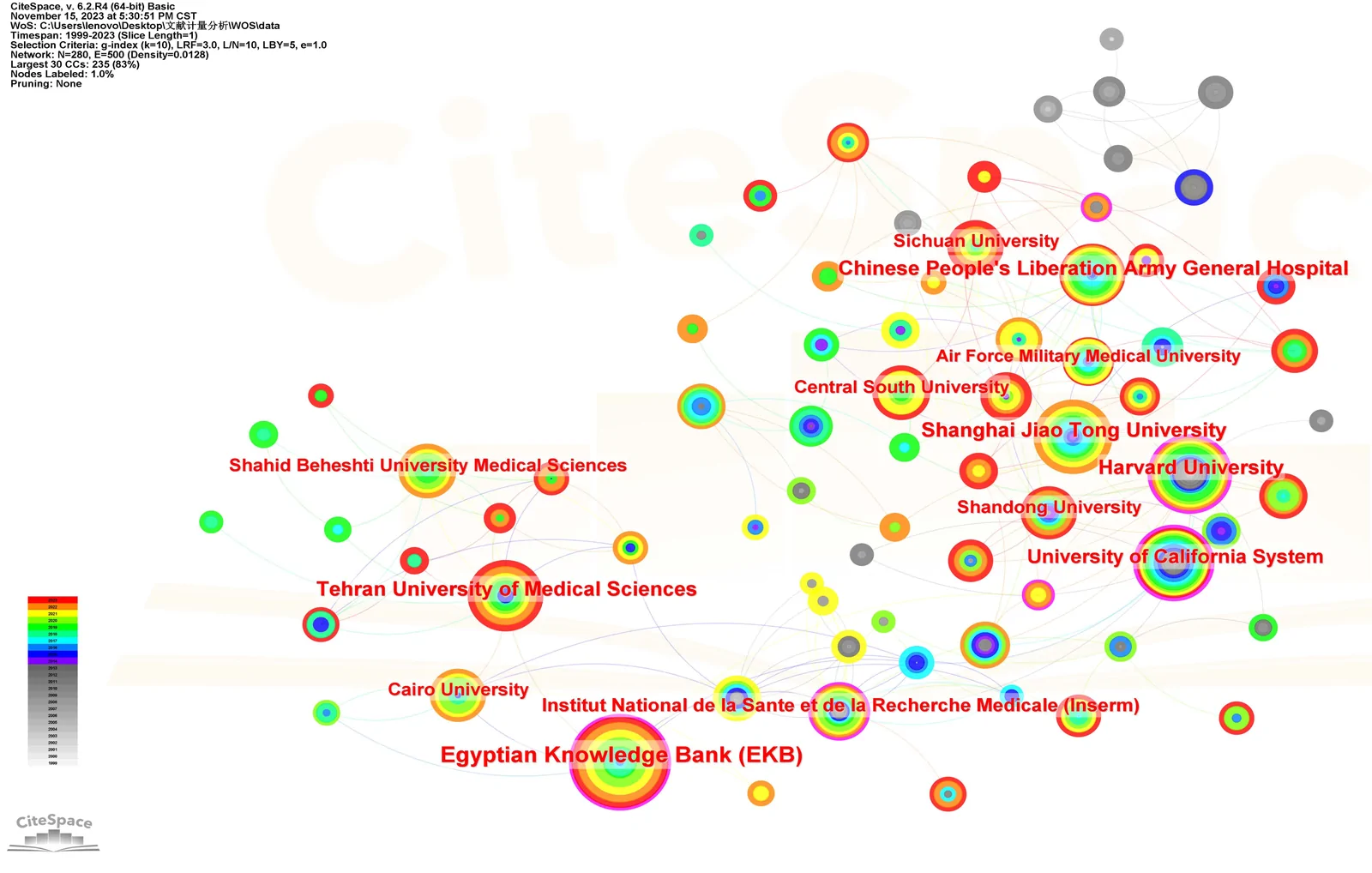
The co-occurrence map of institutions performing research involving the use of MSCs for diabetes treatment.

We used CiteSpace 6.2.R6 to analyze the countries and institutions of the published literature. The results show that 278 institutions from 90 countries (Figs. **6** and **7**) have jointly authored 2912 publications in the past. Table **3** shows that China has the highest number of articles produced N=944), accounting for 32.4% of the total. China is followed by the United States N=694, 23.8%), Italy N=182, 6.26%), Iran N=160, 5.52%), and South Korea N=133, 4.57%]. The number of articles from China and the United States far exceeds that of other countries. Among the top 10 countries with the highest publication volume, 60% are developed countries. Moreover, visualization analysis (Fig. **6**) can provide a clearer view of the frequency, time, and other elements of cooperation among various countries. Among the lines of various countries, there are more blue, purple, and green lines. This indicates that the peak period of cooperation between countries occurred between approximately 2014 and 2020.

Among the 278 institutions, the institution with the most contributed articles is the Egyptian Knowledge Bank (N=87), followed by the Chinese People's Liberation Army General Hospital (N=52), Shanghai Jiao Tong University (N=49), and Harvard University). These top ten institutions are distributed in various countries (Table **4**). As shown in Fig. (**7**), the connections between institutional nodes are very sparse, which indicates that there are few cooperative relationships between institutions. The relatively large number of blue lines also indicates that most of the cooperation between institutions was concentrated in the period from 2015 to 2016.

### 3.4 Authors and Co-Cited Authors

**Fig. (8):**
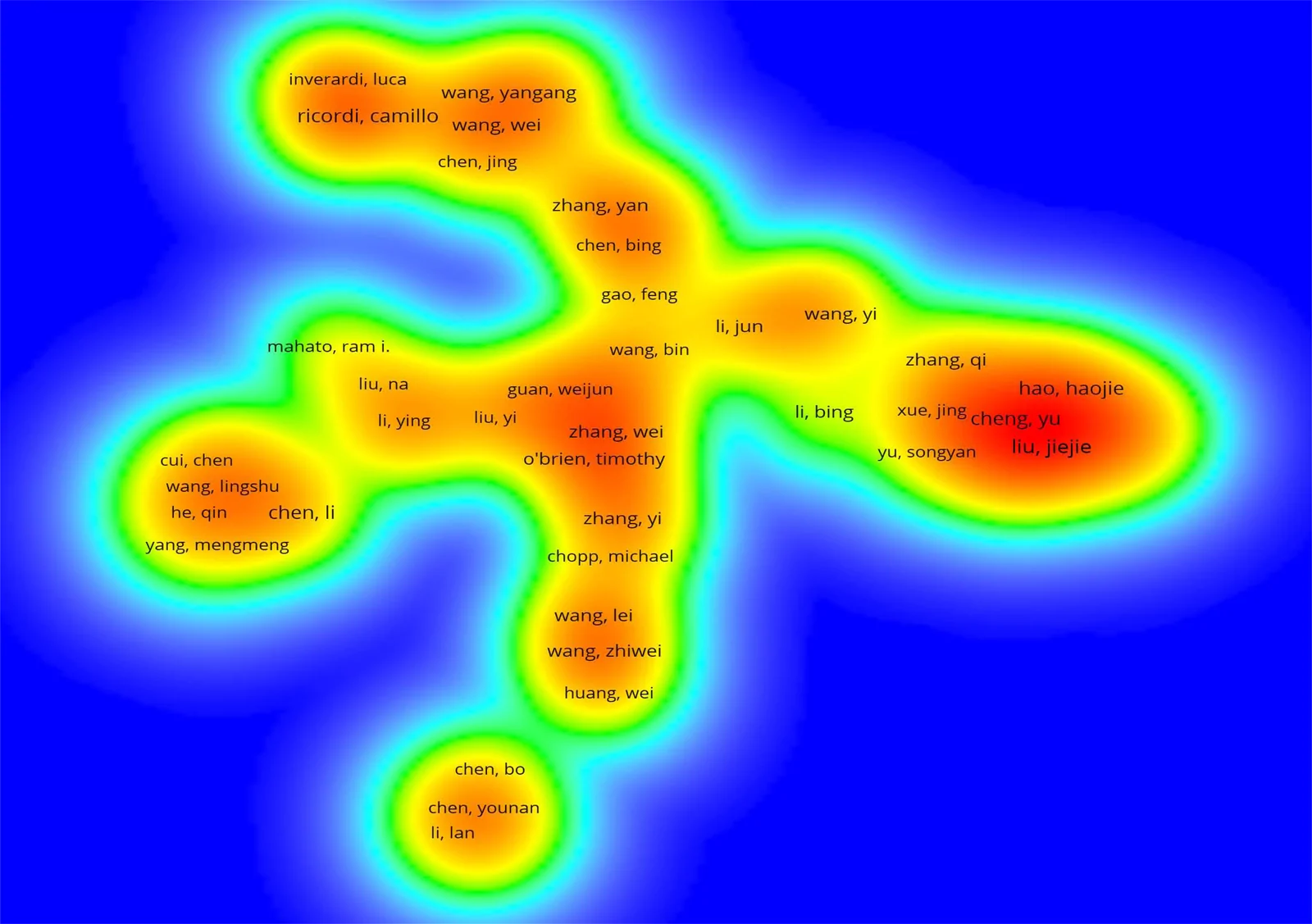
The density map of authors studying the use of MSCs for diabetes treatment, showing the authors with ≥5 publications. **Note:** The size of the word, size of the circle, and opacity of yellow are positively related to the publication frequency.

We identified a total of 15384 authors through VOSviewer. The number of publications by 16 researchers exceeded 10. The author with the most published articles was Liu Jiejie (N=17), followed by Mu Yiming (N=16) and Soleimani Masoud (N=16) (Table **5**). Then, we limited the construction of the density map to a publication volume of more than 5 articles (Fig. **8**). This map clearly shows high-frequency authors who are divided into several closely collaborating author groups, including author groups composed of Liu Jiejie, Han Weidong, Hao Haojie and others, composed of Inverardi Luca, Ricordi Camillo, and others, composed of Wang Zhiwei, Wang Lei, Huang Yan, Huang Wei and others and some other collaborating author groups.

**Fig. (9):**
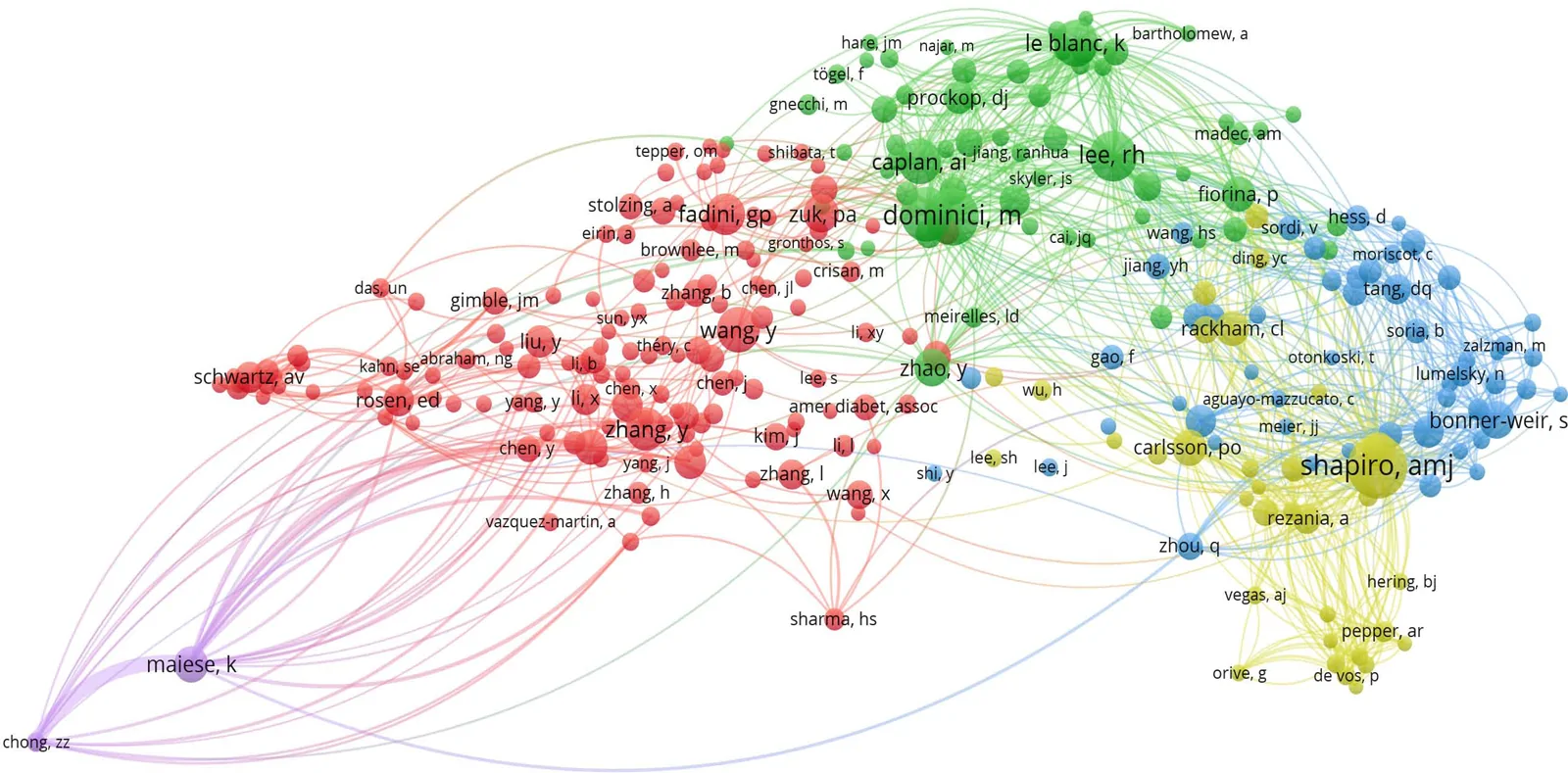
The co-occurrence network and clusters of cocited authors publishing studies related to the use of MSCs for diabetes treatment. **Note:** The size of the word, size of the circle, and opacity of yellow are positively related to the publication frequency.

Among the 86,288 cocited authors, there were 69 whose citation count exceeds 100. The most cited co-authors were Shapiro Amj (n=401), Dominci M (n=340), Pettenger Mf (n=313), Lee Rh (n=286) and Le Blanc K (n=251). The other five cocited authors in the top 10 citation counts ranged from 180 to 250 (Table **6**). We limited the construction of the network to a citation volume greater than 40 times (Fig. **9**). The same color represents the same cluster, which resulted in the authors being grouped into 5 main clusters. This indicates strong cooperation between authors within the same cluster.

### 3.5 Co-Cited Reference and Reference Burst

Cocitation is a research method that measures the degree of closeness between studies. Cocitation refers to the situation in which two or more articles are cited by one or more references, indicating a cocitation relationship between these references. Among the 1478 retrieved cocited references, the top 10 most cited references are displayed in Table **7**. The article, “Multipotent stromal cells from human marrow home to and promote repair of pancreatic islets and renal glomeruli in diabetic” [16] had the highest centrality. Overall, the top ten sources of citations were relatively dispersed.

**Fig. (10):**
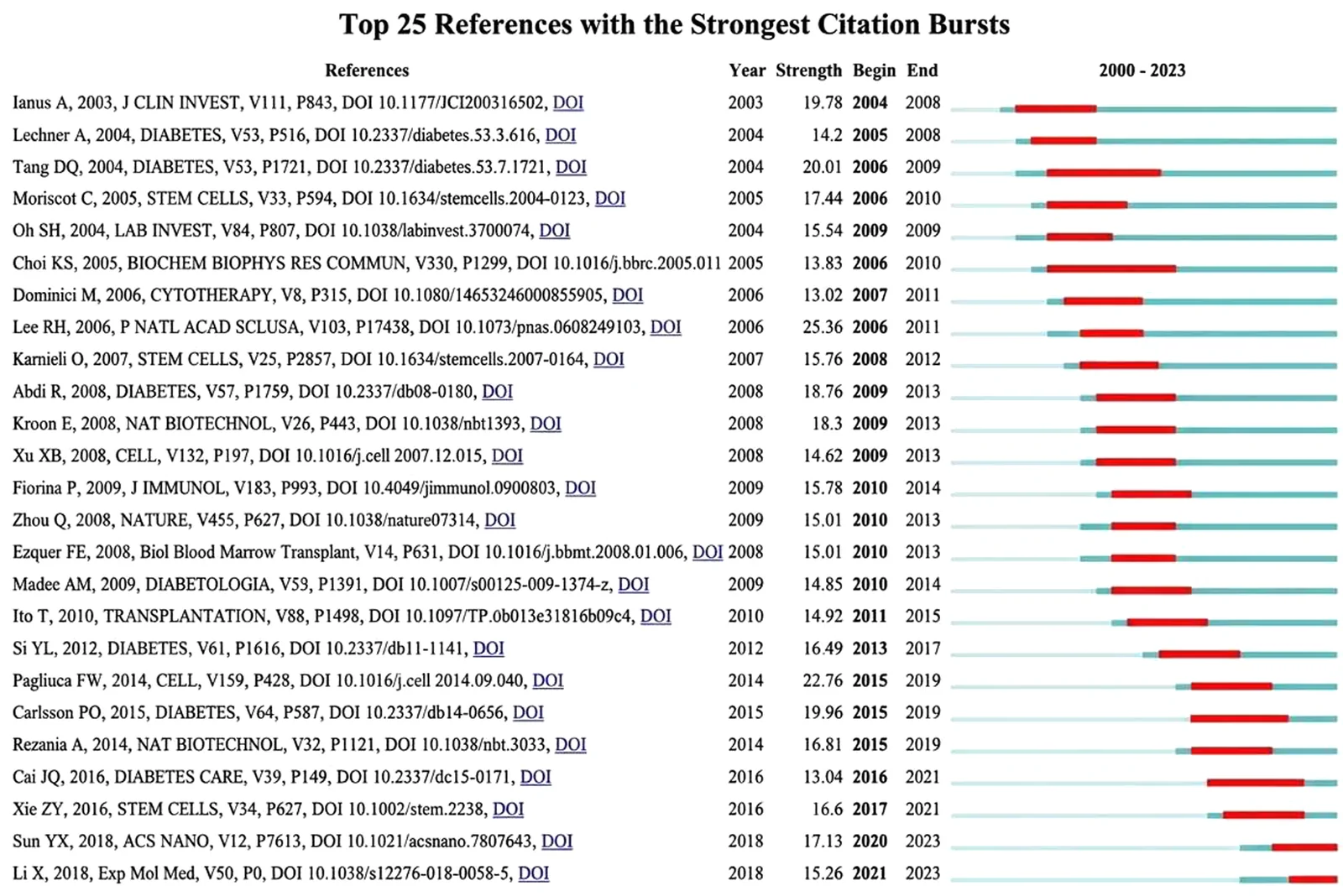
CiteSpace visualization map of the top 25 references with the strongest citation bursts associated with the use of MSCs for diabetes treatment.

Citation bursts are frequently cited within a certain period of time. The duration of the reference burst was ≥2 years, and the top 25 most common outbreaks were identified (Fig. **10**). The most cited burst article was published approximately 2008, and the article with the greatest strength was titled “Multipotent stromal cells from human marrow home to and promote repair of pancreatic islets and renal glomeruli in diabetic NOD/scid mice” (2006) [17]. It is evident that this literature has inspired numerous subsequent studies.

### 3.6 Analysis of Hotspots and Frontiers

#### 3.6.1 Keyword Co-Occurrence Analysis

**Fig. (11):**
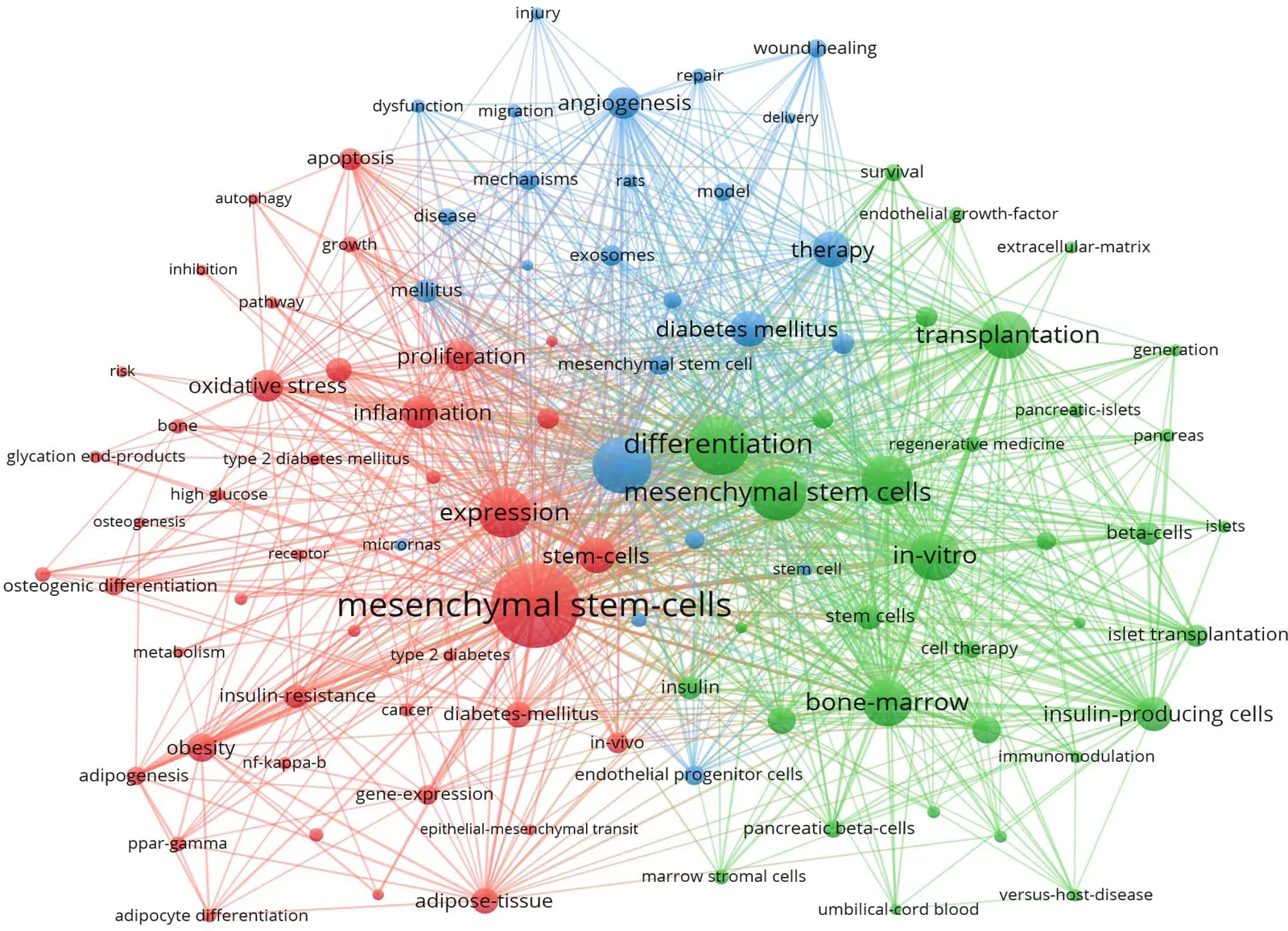
VOSviewer visualization map of keyword clustering analysis related to the use of MSCs for diabetes treatment.

CiteSpace 6.2.R6 was used to analyze keywords, and synonymous keywords such as “mesenchymal stem cells” (N=1850), “diabetes mellitus” (N=750) and “stem cells” (N=431) were excluded from the results (Table **8**). The keywords with high frequency in this study were “expression” (N=564), “transplantation” (N=501), “differentiation” (N=492) and “bone marrow” (N=433), indicating that research in these areas is currently a hot topic. Thus, VOSviewer software was used to conduct keyword co-occurrence analysis, and a graph was constructed with the limit of occurrence times greater than or equal to 50 (Fig. **11**). The co-occurrence network forms three different clusters through different colors, which represent different research directions. The main keywords of the red group were mesenchymal stem cells, expression, inflammation and proliferation. The main keywords of the blue group were diabetes, therapy, angiogenesis and extracellular vesicles. The main keywords of the green group were differentiation, bone-marrow, transplantation and *in-vitro*.

#### 3.6.2 Keyword Time-Cluster Analysis

**Fig. (12):**
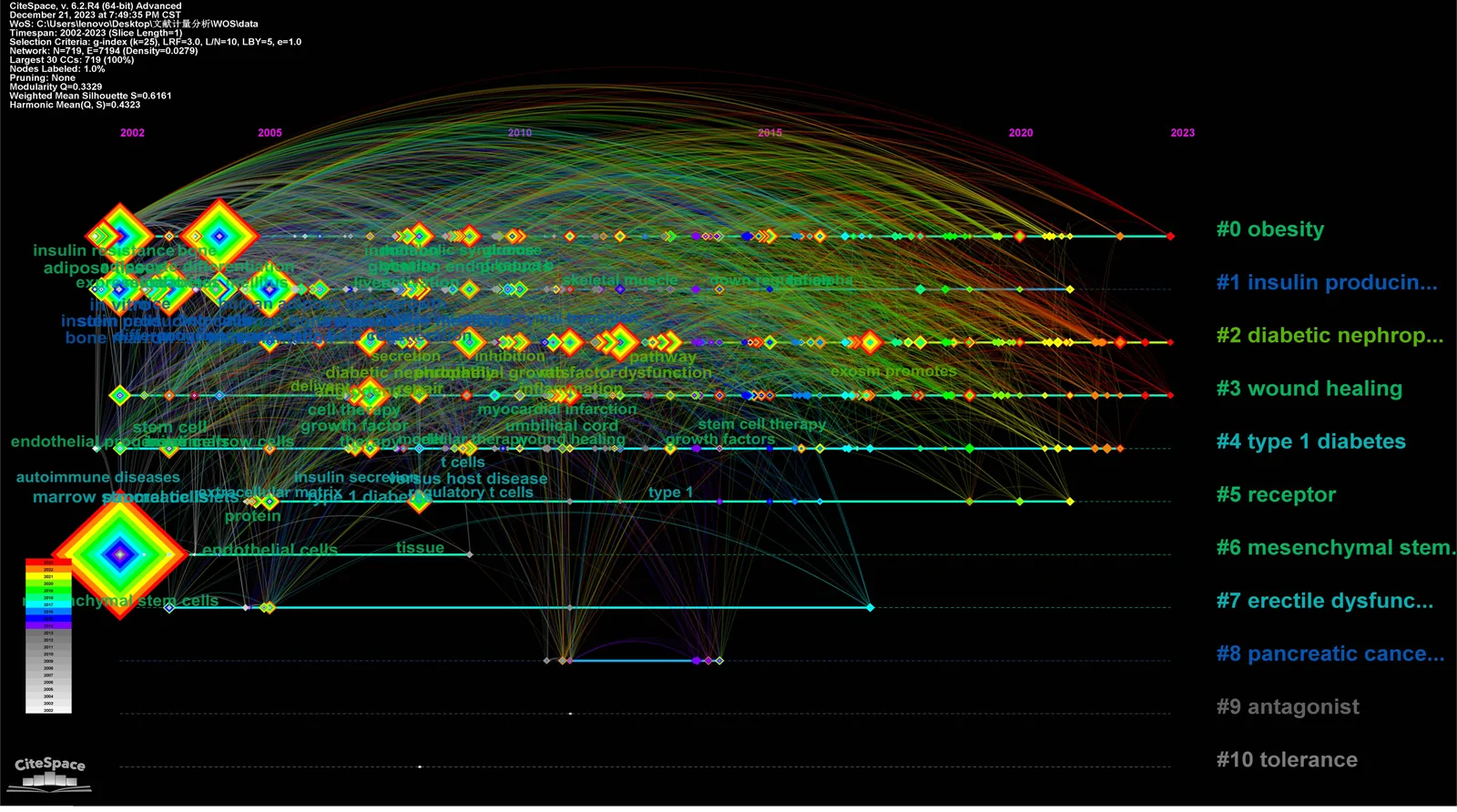
CiteSpace visualization map of the timeline view related to the use of MSCs for diabetes treatment.

The cluster analysis of keywords can better display the knowledge structure of the study, and the combination of time dimensions can better reflect the history of MSC intervention in diabetes. We used CiteSpace 6.2.R6 for time node and cluster analysis (Fig. **12**). A total of 11 clusters were generated, as shown in Table **9**. During the decade from 2000 to 2010, research focused on key words such as “adipose tissue”, “*in vitro*”, “insulin-producing cells”, and “bone marrow”; since 2011, research hotspots have focused on key words such as “oxidative stress”, “extracellular vesicle”, “apoptosis”, and “downregulation”.

#### 3.6.3 Keyword Burst Analysis

**Fig. (13):**
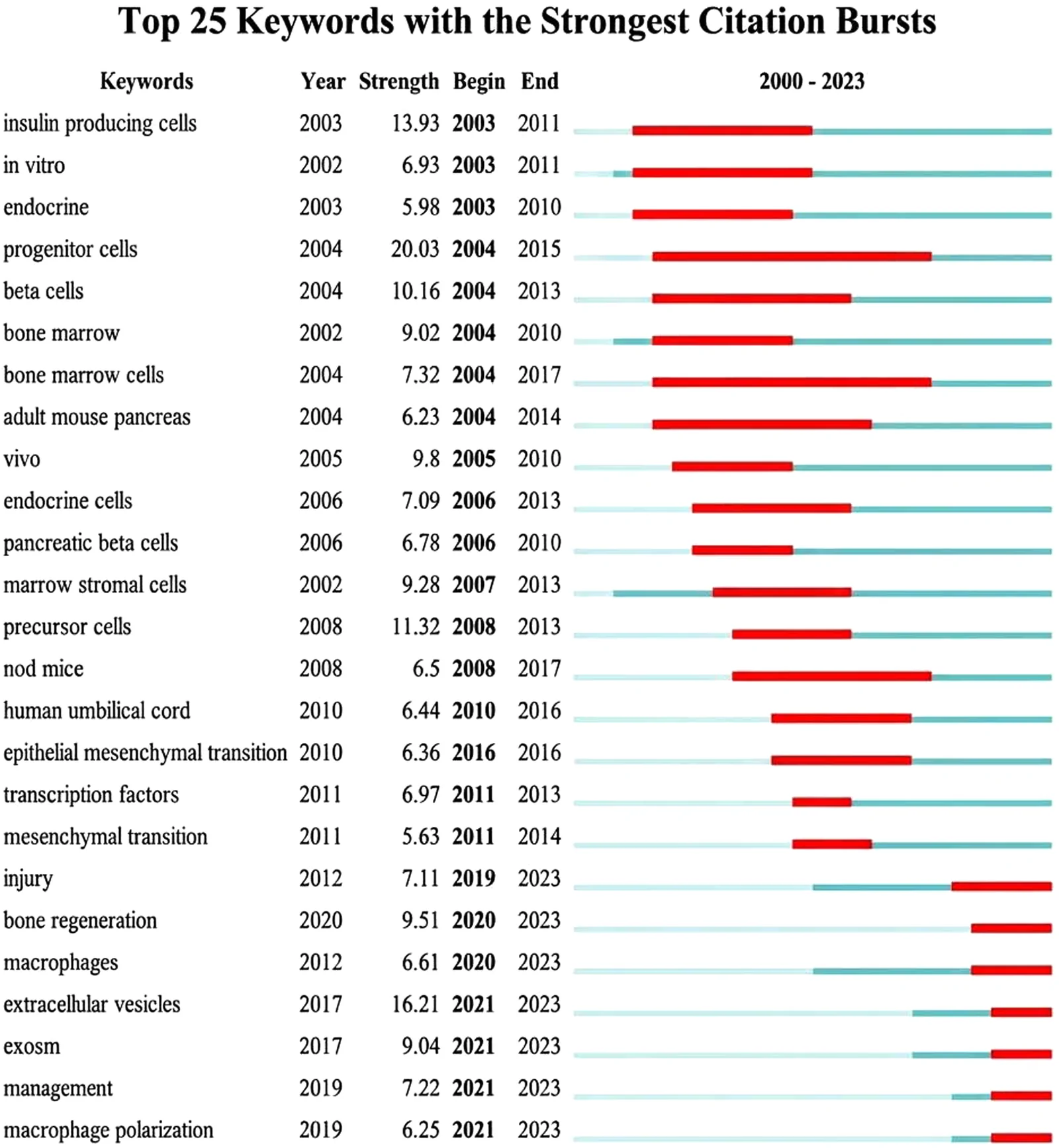
CiteSpace visualization map of the top 25 keywords with the strongest citation bursts associated with the use of MSCs for diabetes treatment.

Keyword bursts are keywords that appear frequently over a period of time, and we identified the top 25 breakout keywords by setting the duration of the bursts to two years or more (Fig. **13**). The keyword with the highest burst strength was “progenitor cells”, which first appeared in 2004, and its outbreak also began in 2004. The keyword with the longest strength time was “bone marrow cells”. The burst time was 13 years from 2004 to 2017. The key words from the outbreak that have continued to this day are “injury”, “one regeneration”, “macrophages”, “extracellular vesicles”, “exosome ”, “management”, and “macrophage polarization”. These findings also show that in the field of MSC intervention in diabetes research, the current most popular direction is the direction represented by these seven keywords.

## 4 DISCUSSION

### 4.1 General Information

The number and trend of published studies each year can reflect the development rate and focus of the research field. As shown in Fig. (**2**), the general trend of published literature on this topic is increasing. Specifically, the number of published studies increased from 2012 to 2014 and from 2018 to 2020, indicating that the field of MSC intervention for diabetes has advanced during these two time periods. During the period from 2012 to 2014, studies have showed that human stem cells can differentiate into islet beta cells *in vitro*, and the function of these cells is similar to that of human primary beta cells after transplantation *in vitro*. The possibility of stem cells intervening in diabetes through differentiation into beta cells has been explored from an *in vitro* perspective [18, 19]. During the period from 2018 to 2020, exosomes from MSCs were shown to protect beta cells from type 2 diabetes mellitus (T2DM) destruction by enhancing insulin signaling and increasing insulin sensitivity and glucose uptake and storage in T2DM rats and insulin-resistant cell models [20]. The exosomal Nrf2 secreted by adipose-derived MSCs can protect endothelial progenitor cells in the high-sugar environment caused by diabetes, thereby reducing the area of foot ulcers complicated by diabetes and improving wound healing [21]. In-depth research on MSC-derived exosomes has revealed new research directions for the treatment of diabetes with MSCs. This has also led to an explosive increase in research in 2018–2020. Overall, MSC treatment for diabetes has good potential for future development.

According to the journal analysis in Table **1**, Stem Cell Research and Therapy (N=112, 3.84%) is the journal that published the most studies about MSC intervention in diabetes. This finding indicates that this field is a very important part of stem cell medical research and represents a future development trend. The analysis of cocited journals (Table **2**, Fig. **4**) indicated that the most frequently cited journal is Diabetes (N=6952 IF=7.7). This was followed by PLoS One (N=4457 IF=3.7), Stem Cells (N=4049 IF=5.2), Proceeding of the National Academy of Sciences of the United States of America (N=3713 IF=11.11) and Nature (N=3037 IF=64.8). Some of the cited literature impact factors were not particularly high, so there is an urgent need for high quality articles in this field to fill this gap.

The analysis of countries/regions and institutions presented in Table **3** indicated that the country with the highest number of published articles is China (N=944), accounting for 32.4% of the total number. This was followed by the USA (N= 694, 23.8%), Italy (N= 182, 6.26%), Iran (N=160, 5.52%), and South Korea N=133, 4.57%). With far more articles coming from China and the USA than from any other country, these two countries are the main drivers of field development, and 60% of the top 10 countries in terms of publication volume are developed countries. Interestingly, when centrality is considered, the USA (centrality=0.48) was far ahead of other countries, indicating that the USA plays a key role in global cooperation among countries. The USA was followed by Italy (centrality=0.19), Egypt (centrality=0.15) and the UK (centrality=0.14), and China, which has published the most papers, has a centrality of only 0.07. These findings indicate that in the process of mutual communication and cooperation, countries such as the United States, Italy, and the UK have played a strong role as bridges, but the international cooperation of China in this field is relatively low. These findings are shown in Fig. (**4**).

Among the top 10 institutions (Table **4**) the distribution of each institution is relatively scattered. There were 3 in China, 2 in the United States and 2 in Egypt, among which Harvard University in the United States (0.23) was the highest. As shown in Fig. (**5**), cooperation among various institutions is relatively sparse. Many institutions lack academic exchanges and do not form networks. Therefore, we suggest that cooperation and connections between various countries and institutions should be strengthened to promote research in the field of MSC intervention in diabetes.

According to the analysis of authors and co cited authors, Liu Jiejie (N=17) was the author with the greatest number of publications. This author was followed by Mu Yiming (N=16), Soleimani Masoud (N=16), Han Weidong (N=14), Ricordi Camillo (N=14) and Bayat Mohammad (N=14). Liu Jiejie is mainly engaged in clinical application research on MSCs [22, 23], among which the clinical progress in the use of MSCs derived from the umbilical cord for the treatment of diabetes has reached phase II trials [24]. Mu Yiming focused more on the mechanism of MSC intervention in diabetes, demonstrating that human umbilical cord MSCs can significantly improve glucose homeostasis and reverse the dedifferentiation of β cells, thereby alleviating the symptoms of T2DM [25]. In addition, he and his team explored the differentiation of various types of stem cells into insulin-producing cells to treat diabetes [26] and the use of MSC combination therapy to promote the regeneration of diabetic islets [27]. Soleimani Masoud focused on immune regulation in type 1 autoimmune diabetes [28, 29] and the role of MSCs in *in vitro* immune regulation and cell protection in the treatment of diabetes [30]. Han Weidong's study focused mainly on cardiomyopathy complicated with diabetes and revealed that MSCs could promote macrophage M2 polarization, thereby improving myocardial damage [31] and myocardial fibrosis [32]. Ricordi Camillo studied the clinical treatment of type 1 diabetes and evaluated the efficacy of umbilical cord MSCs and autologous bone marrow transplantation in the treatment of type 1 diabetes [33, 34]. Bayat Mohammad's research focused on photobiological regulation and the role of human bone marrow MSCs in various ulcers caused by diabetes [35, 36]. Among co cited authors, Shapiro Amj had the highest number of citations (n=401) and was followed by Dominci M (n=340) and Pettenger Mf (n=313).

According to Table **7**, among the top 10 co cited studies related to MSC intervention in diabetes, 4 were related to the induction and differentiation of MSCs or *in vitro* culture into insulin-secreting cells. Among the top 25 references cited with the strongest outbreak, the strongest outbreak was multipotent stromal cells from human bone marrow to promote the repair of pancreatic islets and renal glomeruli in diabetic NOD/scid mice. This study also ranks third in the frequency of co cited literature, indicating that MSCs can be induced to become new cells similar to beta cells and play a role in the islets and glomerulus through homing. This finding opens the research field of stem cell differentiation and *in vivo* homing in the study of MSC intervention in diabetes. However, the mechanisms underlying MSC homing and specific differentiation *in vivo* remains to be explored.

### 4.2 The Hotspots and Frontiers

Keywords are the research theme and core content of a paper. According to the co-occurrence analysis of keywords, we can understand the research hotspots and development of this field. As shown in Table **8**, the most frequently used keywords were “mesenchymal stem cells” (n=1850), “diabetes mellitus” (n=750), “expression” (n=564), “transplantation” (n=501), “differentiation” (n=492) and “bone marrow” (N=433). On this basis, a cluster analysis was carried out, and finally, several clusters were formed. The main topics are reported below.

### 4.3 Obesity and Diabetes

Obesity refers to the abnormal and excessive accumulation of fat, which can be caused by an imbalance between energy intake and long-term energy expenditure [37]. Obesity is associated with an increased incidence of several chronic diseases, including diabetes. Statistically, the relative risk of T2DM in people with obesity is seven times greater than that in people of normal weight, and the risk is greater in overweight people [38]. Moreover, overweight/obesity is also very common in individuals with type 1 diabetes mellitus (T1DM) [39]. Conversely, obesity itself affects people with diabetes. Narime`ne Belhatem reported that obese patients with diabetes had an increased risk of microalbuminuria and macroalbuminuria and tended to have a decreased glomerular filtration rate compared to the general population [40]. This link between obesity and diabetes is often attributed to inflammation [41]. In people with obesity, there is abnormal hypertrophy of fat cells and excessive release of free fatty acids. Researchers have observed an increase in reactive oxygen species (ROS) production in the adipose tissue of mice. Moreover, excess free fatty acids further aggravate inflammation in the surrounding adipose tissue. This results in a stress response in related organelles, among which the stress response in the endoplasmic reticulum is more severe [42-44]. During stress, the transmembrane protein inositol requirement enzyme 1 (IRE1) becomes active and interacts with tumor necrosis factor receptor-related factor 2, which then activates C-Junn-terminal kinase (JNK) and nuclear factor kappa β (NF-Kβ) [45]. These factors can worsen diabetes symptoms by disrupting insulin signaling through the phosphorylation of insulin receptor substrate 1 on serine residues [46].

As MSCs are one of the treatment methods for diabetes, the effects of MSCs on diabetes and obesity have been studied. Shi *et al.* reported that exosomes produced by bone marrow-derived MSCs can reduce the mRNA levels of the proinflammatory factors IL-6 and TNF-α in mouse groin white adipose tissue (iWAT), thereby alleviating obesity and the inflammatory response. Moreover, the protein levels of P-PI3K, P-AKT and GLUT4 in iWAT were increased in obese mice that were continuously given exosomes, alleviating the problem of insulin signaling pathway blockade [47]. In addition, Shree *et al.* treated 3T3L1 preadipocytes, a model of insulin resistance, with adipose-derived MSCs. The authors reported that 3T3L1 preadipocytes did not differentiate into mature adipocytes, indicating that adipose-derived MSCs can intervene in obesity and alleviate insulin resistance [48]. In general, obesity is closely related to diabetes, and MSCs can not only effectively intervene in diabetes but also affect obesity.

### 4.4 Insulin-Producing Cells and Diabetes

The main cause of T1DM is the destruction of islet beta cells, and stem cells can be manipulated to induce differentiation. Therefore, one treatment idea for MSC intervention in diabetes is to induce MSCs to differentiate into insulin-secreting cells to effectively control the disease of T1DM [49-51]. Zhang *et al.* induced the differentiation of bone marine-derived MSCs into insulin-secreting cells by inhibiting COUP-TFI. The authors demonstrated the function of differentiated insulin-secreting cells successively *in vitro* and *in vivo*, indicating the feasibility of this therapeutic approach [52]. Oh *et al.* used extracellular vesicle-mimicking nanovesles (NVs) prepared from a mouse pancreatic β cell line to induce autologous bone marrow-derived MSCs to differentiate into insulin-producing cells. This differentiation can be effectively achieved *in vivo* in mice, and blood glucose levels were controlled in a T1DM model [53].

Jeong *et al.* developed a new 3D culture material based on previous studies. Maltose-binding protein-basic fibroblast growth factor (MBP-FGF2)-immobilized polystyrene plates (PS) [54, 55] were used to construct a platform based on MBP-FGF2, which can enhance the differentiation of human oment-derived MSCs into β cells *in vitro* by upregulating various cell-cell interaction proteins. Moreover, *in vivo* experiments have verified that induced β cells can effectively control hyperglycemia in diabetic mice [56], and this effect has played an important role in the development of this field. However, this method of inducing MSCs to differentiate into insulin-secreting cells or even β cells to treat T1DM is still being investigated in animal experiments at this stage, and no relevant clinical trials have been reported to date. Because its safety and stability cannot be guaranteed, more researchers and institutions need to cooperate in this field. There is hope for a breakthrough in clinical trials.

### 4.5 Diabetic Nephropathy

Diabetic nephropathy (DN) is a microvascular complication of diabetes mellitus and a major risk factor for renal failure in patients with end-stage diabetes mellitus [57]. The main mechanism of DN is related to hyperglycemia, advanced glycation end products, the overexpression of various growth factors, and high levels of ROS [58, 59]. However, there are few effective treatments for DN at present, and the main treatment options are to control hyperglycemia and blood pressure and block the renin-angiotensin-aldosterone system [60]. Another key factor that makes it difficult to effectively treat DN is that the kidney is a terminally differentiated organ with much lower reproductive potential than other organs. Moreover, the kidney is composed of many cells with different functions, which makes it very difficult to regenerate the kidney [61]. With the development of stem cell research, many studies have shown that stem cells can repair kidney injury, which provides a new approach for the treatment of DN.

MSCs have been shown to repair damaged tissues by homing to them. Li *et al.* demonstrated that 48 h after tail vein injection of bone marrow-derived MSCs from DN rats, MSCs were able to effectively home to the glomeruli and tubulointerstitium of the diabetic kidney and exert kidney-protective effects [62]. Dong *et al.* injected urine-derived MSCs into DN rats. They found that MSCs home to the pancreas and kidney but not to the heart or bladder, indicating that MSCs could home to the corresponding organs to play a protective role [63]. Thus, the homing of stem cells tends to occur in more severely damaged tissues.

For MSCs, the mechanism of renal protection and prevention of renal injury is mainly to control local paracrine effects [64]. Renal samples from MSC-treated animals showed an increase in the levels of antiapoptotic, prosurvival and mitogenic growth factors and a decrease in the expression of proinflammatory cytokines [65].

In addition, exosomes secreted by MSCs also play an important role in the treatment of DN. Exosomes are secretory vesicles with a diameter of approximately 40-100 nm that can transmit intercellular information [66, 67]. Zhang *et al.* demonstrated that miR-146a-5p in umbilical cord-derived MSC-derived exosomes was able to act on TRAF6, thereby promoting M2 macrophage polarization and inhibiting renal inflammation in DN rats [68]. Mao *et al.* demonstrated that miR-let-7p in exosomes derived from bone marrow MSCs could act on USP22 and inhibit its expression, thereby inhibiting oxidative stress in the renal tissue of DN rats and alleviating DN symptoms.

In general, there are many mechanisms by which MSCs alleviate DN. MSCs first reach the most damaged organ or tissue by homing and then play a protective role by controlling local paracrine signaling to regulate cytokines. Additionally, exosomes secreted by MSCs from different sources can regulate the immune system or other targets to play a therapeutic role.

## CONCLUSION

Our bibliometric study describes the global patterns and growth trends of mesenchymal stem cell interventions in the field of diabetes from 2000 to 2023. We found that MSC intervention in diabetes patients has received a great deal of attention over time. The number of published papers has steadily increased, and the most influential countries, institutions, and journals in this field have been analyzed. Existing studies have focused mainly on obesity, insulin-producing cells, diabetic nephropathy and differentiation, but there are still some obstacles in this field. Topics such as the clinical application of insulin-producing cells, the specific mechanisms of stem cell homing, and the therapeutic effects of MSCs from different sources may represent the frontiers of future research, and our results may help to identify research hotspots and trends. This study provides a new opportunity for further research and the application of mesenchymal stem cells in diabetes treatment.

## LIMITATIONS

This study is a bibliometric analysis study. The software CiteSpace and VOSviewer cannot replace systematic retrieval, and there may be errors in the processing of information, and the data for this study is as of October 2023 and will not record the latest progress. In addition, all the data in this study were obtained from the literature in the WoS database, so it is inevitable that some of the literature not included in the database was missing. Due to a few subjective factors in the literature screening process, a few effective studies may not be included in the final database. At the same time, some studies involve isolating mesenchymal stem cells from diabetic patients, which is not a treatment, and such articles will inevitably be missed in the process of manual exclusion. Finally, due to the large amount of total data, the minimum frequency of visualization nodes was limited in the process of CiteSpace and VOSviewer visualization analysis, which may reduce the credibility of the study. However, based on the established bibliometric database, this study can help researchers more intuitively understand the research hotspots and development trends in the field of MSC intervention in diabetes.

## Figures and Tables

**Fig. (2):**
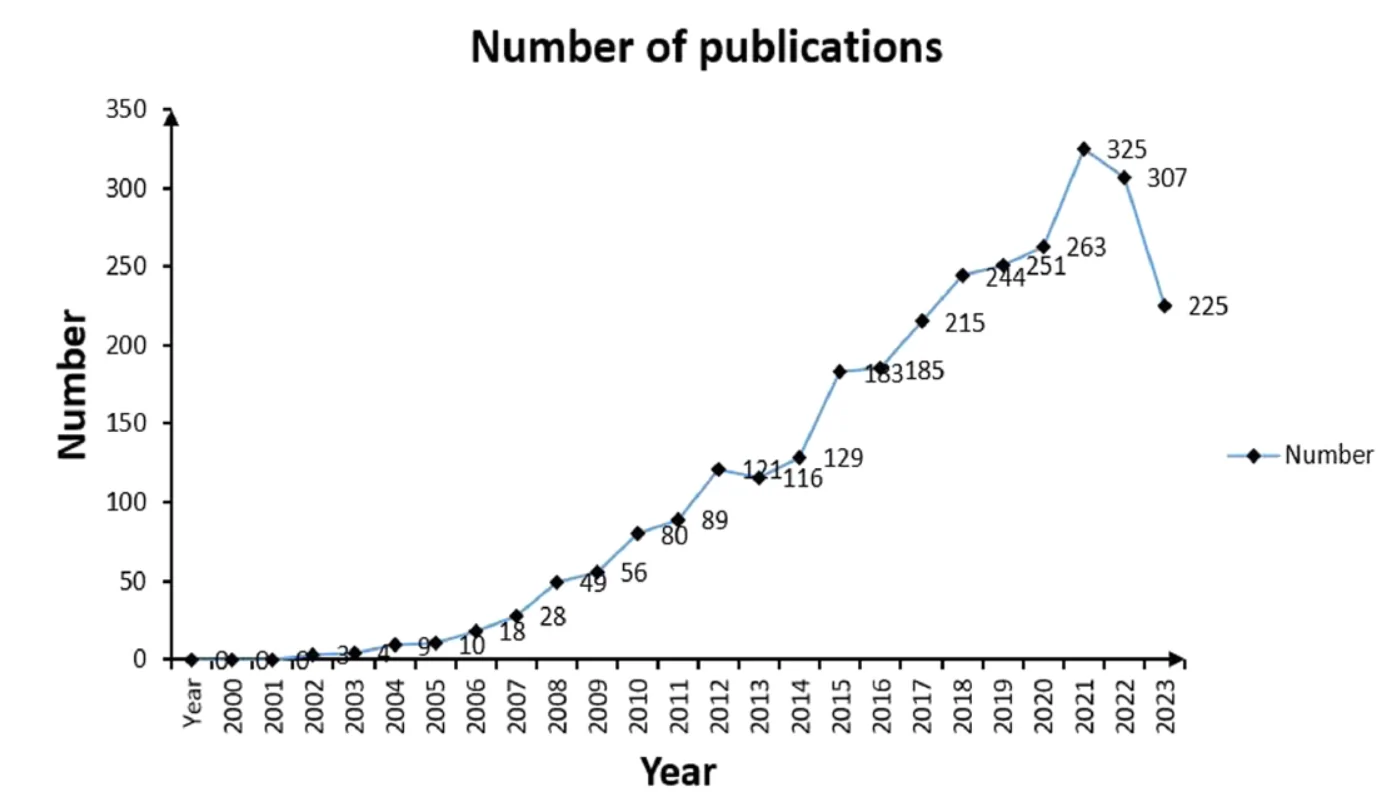
Trends in publications on the use of MSCs for diabetes treatment in the 21^st^ century.

**Table 1:** Top 10 journals related to the use of MSCs for diabetes treatment.

Journal	Count	Percent %	IF(2023)	JCR Division	Country
Stem Cell Research and Therapy	112	3.84	7.5	Q1	England
International Journal of Molecular Sciences	85	2.92	5.6	Q1	Switzerland
Frontiers in Endocrinology	50	1.72	5.2	Q1	Switzerland
PLoS One	47	1.61	3.7	Q2	USA
Stem Cells International	44	1.51	4.3	Q2	USA
Diabetes	37	1.27	7.7	Q1	USA
Stem Cells Translational Medicine	36	1.23	6.0	Q1	USA
Cell Transplantation	35	1.20	3.3	Q2	USA
Stem Cells and Development	34	1.17	4.0	Q2	USA
Cytotherapy	31	0.72	4.5	Q2	England

**Table 2:** Top 10 co-cited journals related to MSCs for diabetes treatment.

Co-Journal	Count	IF (2023)	JCR Division	Country
Diabetes	6952	7.7	Q1	USA
PLoS One	4457	3.7	Q2	USA
Stem Cells	4049	5.2	Q2	USA
Proceeding of the National Academy of Sciences of the United States of America	3713	11.1	Q1	USA
Nature	3037	64.8	Q1	England
Journal of Biological Chemistry	2867	4.8	Q2	USA
Diabetologia	2645	8.2	Q1	Germany
Journal of Clinical Investigation	2468	15.9	Q1	USA
Stem Cell Research and Therapy	2419	7.5	Q1	England
Biochemistry and Biophysical Research Communications	2354	3.1	Q2	USA

**Table 3:** Top 10 countries/regions related to the use of MSCs for diabetes treatment.

Country/Region	Count	Centrality	Percent %	Year
Peoples Republic of China	944	0.07	32.4	2004
USA	694	0.48	23.8	2002
Italy	182	0.19	6.26	2005
Iran	160	0.09	5.52	2010
South Korea	133	0.00	4.57	2005
Japan	128	0.03	4.40	1999
India	119	0.08	4.10	2008
Germany	117	0.09	4.02	2004
England	97	0.14	3.33	2002
EGYPT	95	0.15	3.26	2008

**Table 4:** Top 10 countries/regions related to the use of MSCs for diabetes treatment.

Institution	Count	Centrality	Country	Year
Egyptian Knowledge Bank	87	0.15	Egypt	2014
Chinese People’s Liberation Army General Hospital	52	0.08	China	2013
Shanghai Jiao Tong University	49	0.04	China	2014
Harvard University	48	0.23	USA	2002
Tehran University of Medical Sciences	47	0.08	Iran	2013
University of California System	46	0.22	USA	2003
Institut National de la Sante et de la Recherché Medicale	34	0.17	France	2005
Central South University	31	0.10	China	2006
Shandong University	31	0.01	Egypt	2008
Shahid Beheshti University Medical Sciences	31	0.01	Iran	2017

**Table 5:** The top 10 authors of MSC studies related to diabetes.

**Author**	**Count**	**Country**	**Institution**
Liu Jiejie	17	China	Chinese PLA General Hospital
Mu Yiming	16	China	Chinese PLA General Hospital
Soleimani Masoud	16	Iran	Stem Cell Technology Research Center (Tehran, Iran)
Han Weidong	14	China	Chinese PLA General Hospital
Ricordi Camillo	14	USA	University of Miami Miller School of Medicine
Bayat Mohammad	14	Iran	Shahid Beheshti University of Medical Sciences
Rosen Clifford J	13	USA	The Tufts University School of Medicine
Abraham Nader G	13	USA	Medicine New York Medical College
Hao Haojie	13	China	Chinese PLA General Hospital
Chen Li	13	China	Shandong University Qilu Hospital

**Table 6:** The top 10 cocited authors of MSCs for diabetes treatment.

**Author**	**Count**	**Country**
Shapiro AMJ	401	Canada
Dominci M	340	Italy
Pettenger MF	313	USA
Lee RH	286	USA
Le Blanc K	251	Sweden
Caplan AI	246	USA
Wang Y	239	China
Zhang Y	228	China
Fadini GP	218	Italy
Bonner-Weir S	189	USA

**Table 7:** Top 10 cocited references related to the use of MSCs for the treatment of diabetes (there are 11 studies with the same number of citations).

**Title**	**Year**	**Author**	**Centrality**	**Citations**	**Journal**
Preserved beta-cell function in type 1 diabetes by mesenchymal stromal cells	2015	Per-Ola Carlsson	0.04	51	*Diabetes*
Generation of functional human pancreatic β cells *in vitro*	2014	Felicia W. Pagliuca	0.05	50	*Cell*
Multipotent stromal cells from human marrow home to and promote repair of pancreatic islets and renal glomeruli in diabetic NOD/scid mice	2006	Ryang Hwa Lee	0.20	50	*Proceeding of the National Academy of Sciences of the United States of America*
Human mesenchymal Stem Cell Derived Exosomes Alleviate type 2 diabetes mellitus by reversing peripheral insulin resistance and relieving β-cell destruction	2018	Yaoxiang Sun	0.01	49	*ACS Nano*
Human umbilical cord-derived mesenchymal stem cells elicit macrophages into an anti-inflammatory phenotype to alleviate insulin resistance in type 2 diabetic rats	2016	Zongyan Xie	0.03	44	*Stem Cells (Dayton, Ohio)*
Immunomodulation by mesenchymal stem cells: A potential therapeutic strategy for type 1 diabetes	2008	Reza Abdi	0.06	41	*Diabetes*
Pancreatic endoderm derived from human embryonic stem cells generates glucose-responsive insulin-secreting cells *in vivo*	2008	Evert Kroon	0.03	40	*Nature Biotechnology*
Umbilical cord mesenchymal stromal cell with autologous bone marrow cell transplantation in established type 1 diabetes: A pilot randomized controlled open-label clinical study to assess safety and impact on insulin secretion	2016	Jingquan Cai	0.06	39	*Diabetes Care*
Minimal information for studies of extracellular vesicles 2018 (MISEV2018): A position statement of the International Society for Extracellular Vesicles and update of the MISEV2014 guidelines	2018	Clotilde Théry	0.00	37	*Journal of Extracellular Vesicles*
Reversal of diabetes with insulin-producing cells derived *in vitro* from human pluripotent stem cells	2014	Alireza Rezania	0.02	37	*Nature Biotechnology*
Exosomes from adipose-derived stem cells overexpressing Nrf2 accelerate cutaneous wound healing by promoting vascularization in a diabetic foot ulcer rat model	2018	Xue Li	0.00	37	*Experimental & Molecular Medicine*

**Table 8:** Top 10 keywords related to MSCs for diabetes treatment.

**Keywords**	**Count**	**Centrality**	**Year**
Mesenchymal Stem Cells	2281	0.08	2002
Diabetes Mellitus	750	0.04	2004
Expression	564	0.04	2002
Transplantation	501	0.04	2005
Differentiation	492	0.03	2003
Bone Marrow	433	0.05	2002
*In Vitro*	428	0.07	2002
Therapy	262	0.03	2007
Oxidative Stress	228	0.01	2012
Adipose Tissue	212	0.05	2002
Insulin Producing Cells	209	0.03	2003
Proliferation	202	0.01	2009
Insulin Resistance	195	0.02	2002
Progenitor Cells	183	0.02	2004
Angiogenesis	160	0.03	2007
Activation	157	0.02	2008
Beta Cells	152	0.02	2004
Inflammation	145	0.02	2011
Tissue	124	0.02	2008
*In Vivo*	121	0.03	2002

**Table 9:** Top 10 keyword clusters related to the use of MSCs for diabetes treatment.

**Cluster ID**	**Cluster**	**Size**	**Mean (Year)**	**Keywords**
#0	Obesity	168	2012	Diabetes mellitus; metabolic disease; bone marrow ;nuclear translocator-like protein-1; muscle aryl hydrocarbon receptor.
#1	Insulin Producing Cells	148	2007	Mesenchymal stem cells; insulin-producing cells; cell therapy; renal repair; acute kidney injury.
#2	Diabetic Nephropathy	135	2016	Mesenchymal stem cells; oxidative stress; green tea; animal models; noncoding RNA.
#3	Wound Healing	120	2014	Diabetes mellitus; stem cell therapy; peripheral artery disease; peripheral blood mononuclear cells; bone marrow mononuclear cells.
#4	Type 1 Diabetes	80	2011	Mesenchymal stem cells; islet engraftment; animal models; local engraftment; intrinsic cross-linking.
#5	Receptor	24	2010	Mesenchymal stem cells; bone marrow; osteogenic differentiation; osteoclast induction; arnt-like protein.
#6	Mesenchymal Stem Cells	16	2004	Mesenchymal stem cells; human bone marrow; human amnion-derived mesenchymal stem cells; regulatory T cells; controlled trial.
#7	Pancreatic Cancer	13	2008	Pancreatic cancer; ovarian cancer; endometrial cancer; breast cancer; prostate cancer.
#8	Erectile Dysfunction	13	2012	Erectile dysfunction; vascular regenerative therapy; endothelial progenitor cells; streptozotocin-induced diabetic rats; surrogate beta cells.
#9	Antagonist	1	2011	Autoimmunity; islet; tolerance; T cells; immunomodulation.
#10	Tolerance	1	2008	Antagonist; fibrosis; lysophosphatidic acid; receptor.

## Data Availability

All remaining data and materials are available from the authors upon reasonable request.

## LIST OF ABBREVIATIONS

DN: Diabetic nephropathy
iWAT: Groin White Adipose Tissue
IRE1: Inositol Requirement Enzyme 1
JNK: Junn-Terminal Kinase
MSC: Mesenchymal Stem Cell
NVs: Nanovesles
NCDs: Noncommunicable Diseases
NF-Kβ: Nuclear Factor Kappa Β
PS: Polystyrene Plates
ROS: Reactive Oxygen Species
T1DM: Type 1 Diabetes Mellitus
T2DM: Type 2 Diabetes Mellitus
WoS: Web of Science
